# Transplanting an Eye

**Published:** 1887-06

**Authors:** 


### TRANSPLANTING AN EYE.

Among the feats illustrating the wonderful progress of modern surgery is the transplantation of eyes. Upon rabbits transplantation has proven eminently successful, but upon man it has never been given a fair trial until the beginning of this year. The experiment was then tried in this city and, so far as transplantation is concerned, was a perfect success. The eye of a live rabbit was placed in the empty conjunctival sac of man, the muscles, nerves and tissues united, and the eye actually became a part of the human organism. However, as a whole, the experiment was a failure, arising from the difficulty encountered during the healing period. This difficulty is attributed to the insufficient vitality of the rabbit's eye. As the muscular power of man is greater than that of the rabbit, so the muscular action of the eye is greater, and this action the transplanted eye was unable to withstand.

One naturally inquires why the rabbit's eye was used. Because the rabbit's eye, although somewhat smaller, comes nearer in resemblance to the human eye than that of any other animal. The arrangement of muscles and blood-vessels in the rabbit is identical with that of the human eye. This is the first time the experiment was ever tried under favorable conditions, and the fact that it will probably never be attempted again makes the case a remarkable one. Of course, if the eyes of man could be transplanted it would be within bounds to anticipate successful results, but as there might be objections to this, the feasibility of transplantation in man may be regarded as practically and finally settled.

The experimental operation was performed upon Mr. Charles Alfred

Williams, City Editor of the Minneapolis *Tribune*, by Dr. Charles H. May, instructor in ophthalmology at the New York Polyclinic and clinical assistant in the same department at the New York College of Physicians and Surgeons. He was assisted by Dr. E. Gruening, also a prominent oculist of this city, and Dr. Wilmer, of Mt. Sinai Hospital, in the presence of a half-dozen students of the institutions named, at No. 119 East Fifty-ninth Street.

The operation was originated and first attempted by the eminent French oculist, Dr. Chibret, of Paris, on May 4, 1885, and first recorded in the Parisian *General Review of Ophthalmology* on the 31st of the same month. In reviewing the operation, Dr. Chibret strongly argued against further attempts upon human beings until the success of the operation had been proven by repeated trials upon the rabbit. However, his advice was not closely followed, and no less than four failures were recorded within five months following the report of the first case. Success in any of these cases would have been considered a miracle, for in no instance had any previous experiments been attempted. In one case a dog's eye was used.

Quite naturally these experiments and the subsequent reviews in the medical journals attracted much attention. If the eye of a rabbit could be used to replace a defective eye in man, it would establish a new era in ophthalmology. The French artificial, or glass eyes, would be laid aside. Instead of being bothered by them, the unfortunate could be supplied with a living eye, operated by the same muscles and moving in perfect unison with his uninjured eye, and so nearly alike in size and color that only the most experienced oculist could distinguish the difference. Sight alone could not be transferred. The importance of the operation, if successful, is thus made manifest, without even considering the expense attached to replacing glass eyes.

It remained for a New York oculist to carry out the suggestions of Dr. Chibret. Dr. Chas. H. May determined to thoroughly test transplantation upon rabbits. He began his experiment January 30, 1886, and during the next two or three months operated upon no less than twenty-four rabbits. Notwithstanding the trouble encountered in keeping the bandages upon the animals, the results obtained under favorable and unfavorable circumstances were carefully noted, the favorable conditions were taken advantage of, and his skill in the manipulation of the instruments increasing as the experiments progressed, success was at last attained. Having now acquired the knowledge of what was necessary, and almost absolute accuracy in technique, the transplantation of the eye of one rabbit to the head of another was successful in four other cases. The rabbits lived, and, although blind in one eye, this defect could only be discovered by shaking something before the transplanted orb and observing that it did not move.

The New York *Medical Record* of May 29th, 1886, contained the review of these experiments. In this Dr. May said: "The results of the operations certainly justified trials upon the eyes of man—at least the transplantation of the rabbit's globe into the human conjunctival sac," and added, in conclusion, that "in no case was there any rise of temperature or any apparent interference with the general health, or the slightest implication of the sound eye," and that in case of failure a glass eye could be easily substituted. The experiments had now been carried so far that the day was eagerly looked forward to when the feasibility of the operation could be tested upon man, and the success or failure established under such conditions as would forever settle the question.

The opportunities for making the decisive trial were not great. There were any number of one-eyed men willing to try it if assured of positive success, but to submit to an experiment, and a delicate and difficult one at that, was an entirely different thing. In consequence, the brightness of the prospect for making the test began to grow decidedly dim. About this time a review of the article in the *Medical Record* chanced to fall under the eye of Mr. Williams. He had lost the sight of his right eye when a lad of thirteen, in a patriotic outburst of youthful enthusiasm attendant upon the celebration of the Fourth of July. Mr. Williams is a young man under thirty, with the excellent physique, good habits and abnormal nerve that usually distinguish newspaper men among the ordinary mortals they are compelled to mingle with. He at once concluded to give the rabbit a chance. He notified the New York physicians of his desire to assist the cause of science, and arrived in this city on the 27th of January last. His eye was carefully examined, and it was found that the conditions were favorable for making a test case.

The two weeks prior to the arrival of the patient had been occupied in attempting to secure a rabbit with hazel eyes of the largest size. A fine specimen of the variety known as the Belgian hare was finally selected. The eyes were just a shade darker, and only two-twenty-fifths of an inch smaller than the uninjured one of the patient. The time of the operation was fixed for 2 o'clock on the afternoon of February 1st. Every precaution had been taken to insure success if it were possible. The physicians had even tested their knowledge and skill upon as many fresh cadavers as could be procured at the college dissecting room. The operation was performed without a slip, with the result already made known.

The rabbit was placed upon a small table and the patient upon a lounge a few feet distant. The former was then subjected to the influence of ether, five ounces of the drug and ten minutes' time being required to effect the result. The ether was then administered to Mr. Williams. While this was taking effect the right eye of the rabbit was being operated upon. The globe of the eye was rapidly and carefully separated from the conjunctiva, or sac holding it, the muscles severed and the eye left in the conjunctival sac only attached to the optic nerve. This was done to keep the eye alive until the instant of transplantation. The surgeons next turned their attention to the patient. A spring speculum was introduced under the lids of the right eye to give perfect freedom for the operation. The globe was separated from the conjunctiva and black silk stitches or sutures were carefully passed through either side. As the muscles were severed the four principal or superior ones were also held by the silk sutures. The optic nerve was now the only connecting link. The crisis was now at hand. Owing to the fact that the optic nerve extends directly from the rear of the eyeball it is impossible to see or get hold of this nerve while the globe of the eye remains in the conjunctival envelope. To overcome this difficulty Dr. May had invented a combination forceps and needle especially for the occasion, with an internal concave circumference corresponding to the size of the optic nerve, fitted with fine teeth, with an opening one-eighth of an inch above the termination to admit the passage of a threaded needle, this latter being delicately rounded, curved almost at right angles, the point flattened from side to side and provided with a fine opening. With the special forceps the optic nerve was grasped close to the eyeball and held while it was transfixed by the needle passing through it with a catgut suture. The edges of the conjunctiva, the four principal muscles and the optic nerve were now held in place by the ends of these sutures or threads, while the eyeball was quickly removed.

The optic nerve of the rabbit's eye was now severed, a catgut suture passed through the portion attached to the eyeball and the eye transferred to its new home in the vacant conjunctival sac of the patient. One minute's time was occupied in the transfer. The two optic nerves were drawn closely together by means of the catgut sutures, then firmly tied. The silk sutures holding the muscles were treated in a similar manner, then the conjunctiva was drawn around the rabbit's eye and stitched to the margin of the conjunctiva left surrounding the transplanted globe. The transplantation was now complete. Ten stitches were used in the operation, all of black silk thread excepting those passing through the optic nerve, and these being of catgut because they were to be absorbed by the nerve, while the silk would have to be removed.

After the operation both eyes were closed, the lids of each covered with white vaseline, compresses of cotton placed upon them and then tightly bandaged. The patient was then placed in bed, and applications of hot water placed upon the transplanted eye every few minutes, for the purpose of aiding by heat and moisture the adhesion of the several parts. It was nearly dark when the patient recovered from the influence of the ether, having remained unconscious over two hours. Beyond a dull ache and soreness about the right side of the head he had no personal knowledge of what had taken place. Everything worked like a charm. There had not been a displacement or a slip. The operation occupied one hour and a quarter. On the second day the cornea or surface of the transplanted eye showed a slight haziness, much less than had been anticipated, and this continued throughout the experiment. On the fourth day, on the removal of the bandages—which, by the way, were changed three times a day—it was discovered, by moving the uninjured eye, that the severed parts had grown together, and that the rabbit's eye was actually a part of the human being. The silk sutures were thereupon removed. The union was complete, the transplantation an accomplished fact.

The eyelids were considerably swollen, and the eye still sore. To allay the swelling, remove the soreness and restore the eye to its normal condition was now the object. The improvement was steady and encouraging. On the sixth day the uninjured eye was relieved of the bandage and the room darkened. On the morning of the eighth day the prospects of success were still bright, but on the evening of the same day there was a change. A slight abrasion at the lower edge of the cornea was observed.

The following morning the patient remarked that the eye "felt as though it had got loose from the dock and was floating around in mid-stream." The removal of the bandage revealed, that during the night the greater part of the iris, or coloring matter of the eye, had escaped through the opening in the cornea. The experiment so far as the retention of the perfect rabbit's eye was concerned, was a failure. The eye, however, remained, but in a colorless condition. The question of healing the abrasion or removing the eye was now considered. It was found that to do the first would further disfigure the eye by leaving an irregular scar across the surface of the eye, so it was decided to remove the rabbit's eye and prepare for the insertion of a glass eye.

The second operation was submitted to on the afternoon of the ninth day without the use of ether, cocaine being used as a local anæsthetic. This lasted twenty minutes. All the muscles were found perfectly united, and the optic nerve had grown together and completely absorbed the cat-gut sutures. The conjunctival sac was next drawn over the optic nerve, sutured thereto, the bandage again put in place after the use of more vaseline, and the patient permitted to leave his bed and room during the process of healing, which must take place before the insertion of the artificial eye. The rabbit, not having to undergo the transplantation, quickly recovered from the operation, and is now in perfect condition for the reception of his glass eye.

Thus ended the first and only test case of this kind. The operation has been considered of less consequence than its importance merited, through having been confounded with the transplantation of the cornea of a rabbit's eye to the globe of the human being. This latter operation has been known for over thirty years, holds a place in the text books, and has been successfully performed quite a number of times, notably in Berlin and Vienna. But it is a very simple operation when compared with the one described in the foregoing article.—*N. Y. World.*

